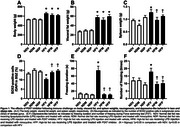# The administration of purinergic receptor P2X7 inhibitor alleveates depressive‐like behavior in obese and lean rats with immune challenge

**DOI:** 10.1002/alz70855_098556

**Published:** 2025-12-23

**Authors:** Titikorn Chunchai, Hiranya Pintana, Patcharapong Pantiya, Busarin Arunsak, Kittikhun Lapto, Suriphan Donchada, Oangkana Pengain, Chanon Kunasol, Nipon Chattipakorn, Siriporn C Chattipakorn

**Affiliations:** ^1^ Neurophysiology Unit, Cardiac Electrophysiology Research and Training Center, Faculty of Medicine, Chiang Mai University, Chiang Mai, Thailand; ^2^ Center of Excellence in Cardiac Electrophysiology Research, Chiang Mai University, Chiang Mai, Thailand; ^3^ Cardiac Electrophysiology Unit, Department of Physiology, Faculty of Medicine, Chiang Mai University, Chiang Mai, Thailand; ^4^ Department of Oral Biology and Diagnostic Sciences, Faculty of Dentistry, Chiang Mai University, Chiang Mai, Thailand

## Abstract

**Background:**

Chronic high‐fat diet (HFD) consumption and lipopolysaccharide (LPS) injection prime microglia, enhance inflammatory responses, decrease neurogenesis, resulting in depressive‐like behavior. It has been reported that LPS stimulates adenosine triphosphate production from immune cells and dead cells, activating microglia via the purinergic receptor P2X7. However, the effects of P2X7 inhibitors on body weight, visceral fat, spleen weight, neurogenesis, and depressive‐like behavior in obese and lean rats under immune challenge remain unclear.

**Method:**

Thirty‐six male Wistar rats were fed either a normal diet (ND) or HFD (59.28% energy from fat) for 12 weeks. Each group was divided into three subgroups receiving either normal saline solution, minocycline (45 mg/kg, two doses, every 12 hours, intraperitoneal (i.p.) injection), or P2X7 inhibitor (JNJ‐55308942, 30 mg/kg, a single dose, orally). To induce immune challenge, all rats received LPS (500 µg/kg, a single dose, i.p.) after their assigned tretments for an hour. Depressive‐like behavior was assessed 24 hours after immune challenge, followed by euthanasia.

**Result:**

All HFD‐fed rats equally increased body and visceral fat weights (*p* <0.05, Figure 1A‐B). Spleen weight decreased in ND‐fed rats treated with minocycline, when compared to ND‐fed rats treated with vehicle, suggesting suppression of inflammatory responses by minocycline in lean rats. In addition, HFD‐fed rats receiving vehicle also increased spleen weight, which was decreased by both treatments, indicating the anti‐inflammatory effect of both treatments in obese rats receiving an immune challenge (*p* <0.05, Figure 1C). Moreover, SOX2‐positive cells in the subgranular zone of the dentate gyrus was reduced in HFD‐fed rats treated with vehicle, suggesting a decrease in neurogenesis. Interestingly, the number of neurogenesis was restored in HFD‐fed rats treated with minocycline or a P2X7 inhibitor (*p* <0.05, Figure 1D). In addition, the number and latency of freezing time in the forced swim test increased in HFD‐fed rats treated with vehicle, which were attenuated in HFD‐fed rats treated with minocycline or P2X7 inhibitor (*p* <0.05, Figure 1E‐F).

**Conclusion:**

In addition, HFD‐fed rats receiving vehicle also increased spleen weight, which were decreased by both treatments, indicating anti‐inflammatory effect of both treatments in obese rats receiving immune challenge (*p* <0.05, Figure 1C).